# Food hygiene and food safety practices of households in a township north of Tshwane, Gauteng

**DOI:** 10.4102/hsag.v28i0.2346

**Published:** 2023-10-17

**Authors:** Mashudu Manafe, Reno E. Gordon, Lindiwe J. Ncube

**Affiliations:** 1 Departments of Human Nutrition and Dietetics, Schools of Health Care Sciences, Sefako Makgatho Health Sciences University, Pretoria, South Africa; 2 Department of Hospitality Management, Faculty of Hospitality and Tourism Management, University of Mpumalanga, Mbombela, South Africa

**Keywords:** food safety, food hygiene, food handlers, Tshwane, households, foodborne illnesses

## Abstract

**Background:**

Foodborne illness is still a major public health concern worldwide. Most cases of foodborne illness can be traced to the home. The food safety practices of food handlers in the household are an important determining factor in food safety at the household level.

**Aim:**

To assess the food safety practices of food handlers in households in Ga-Rankuwa, Tshwane.

**Setting:**

The study was conducted in zones 1–9 and zone 16, as well as extension 25 of Ga-Rankuwa, Tshwane.

**Methods:**

A quantitative descriptive study design was used for which a structured questionnaire was administered among 339 food handlers. Descriptive and inferential statistical analysis was performed in STATA 13.1.

**Results:**

The majority of food handlers reported always washing their hands before (81%) and after handling raw meat, chicken or fish; the majority of food handlers (69%) reported always washing preparation surfaces and utensils with clean, soapy water after handling raw meat, chicken or fish. Less than half (45%) of food handlers reported never thawing frozen meat, chicken or fish in a bowl of cold water.

**Conclusion:**

The food handlers reported appropriate food safety practices regarding hand washing and food preparation surfaces and utensils. However, their practices regarding the storage of meat, chicken and fish were inappropriate.

**Contribution:**

This study builds on the existing body of literature on the food safety practices of food handlers. Moreover, the study findings can serve as a basis for the development of interventions to ensure food safety at a household level.

## Introduction

Food hygiene and food safety remain as critical public health challenges in both developed and developing countries. According to the World Health Organization (WHO), five main aspects to safer food include keeping it clean, separating raw and cooked food, cooking thoroughly, storing food at safe temperatures, and using safe water and raw materials (WHO 2006). This is to ensure the presentation of safer food, optimal health and prevention of foodborne illnesses. Foodborne illness affects 1 out of every 10 people worldwide (WHO 2022a). Takanashi et al. (2009) reported that 70% of diarrhoea cases in developing countries are caused by pathogens transmitted through food. Sub-Saharan Africa accounts for 53% of all foodborne illness and 75% of related deaths globally (Preneuf & Morales 2018). Thus, unsafe food creates a vicious cycle of diarrhoea and malnutrition, particularly affecting elderly, young children and the immunocompromised (WHO 2022a). The majority of cases of foodborne disease are reported to originate from the home (Bloomfield & Nath 2013). In Africa, children under the age of 5 years and people who live in low-income homes are most at-risk to foodborne illnesses (Newell et al. 2010).

A possible cause of foodborne illness is food contamination which can result from poor food safety knowledge and practice of food handlers in the home, which is the main location for foodborne illness outbreak Byrd-Bredbenner et al. (2013). Both Ehuwa, Jaiswal and Jaiswal (2021) and Preneuf and Morales (2018) reported that at least 10%– 20% of foodborne illness outbreaks were due to contamination by the food handlers. An example of such outbreak was documented among paediatric patients attending outpatient clinics in Ethiopia; parasites (*Salmonella* and *Shigella*) were found and this was attributed to poor food safety practices (Ayalew, Amare & Bthatesfa 2013). The factors which can lead to food contamination are improper handwashing, not keeping utensils and kitchen surfaces clean, and improper thawing of meat, chicken and fish (Ganta & Kadeangadi 2019). Correct measures of handwashing are important for ensuring food safety in the home.

Available published literature is mainly on the food safety practices in hospitals and schools (Sibanyoni, Tshabalala & Tabit 2017; Teffo & Tabit, 2020). In one study which assessed the food safety practices of households in KwaZulu-Natal, South Africa, 24% of food handlers stated that meat should be uncovered while refreezing and 30% stated that meat should not be refrozen (Mkhungo, Oyedeji & Ijabadeniyi 2018). The majority of food handlers (> 70%) in the KwaZulu-Natal study did not know the appropriate cold storage temperatures. These are critical areas in food safety where there are knowledge gaps, and these areas can result in severe foodborne illness for household members.

The WHO’s 2022–2030 strategic plan prioritises the reduction of foodborne illnesses, with the home playing an important role in this strategy (WHO 2022b). The South African government is focused on food safety, with increased regulation of food handling and processing, sales and exports. Additionally, no previously published study has looked at the food safety practices of household food handlers in Gauteng. Thus, the study aims to assess the food hygiene and food safety practices of households in Ga-Rankuwa, Tshwane. The current study addresses this research gap and the findings could have implications for future interventions for food safety in South Africa.

## Research methods and design

### Study design

A quantitative descriptive study design was used to describe the food safety and food hygiene practices of households in Ga-Rankuwa, Tshwane.

### Study population and sampling strategy

The target population was households in Ga-Rankuwa from 11 zones, namely zones 1–9 and zone 16, as well as extension 25 with a total of 7700 households. From this, the sample size of 367 was calculated using the online Raosoft sample size calculator with a 5% margin of error, 95% confidence level and 50% response distribution. Proportionate sampling was used to select number of households from each of the 11 zones. A list of households was obtained from the Ga-Rankuwa Municipality Offices. Probability systemic sampling was used, in which every fifth house was selected, until the sample size of 367 households was met (Leedy & Omrod 2009). Child-headed households were excluded from this study as were selected households in which there were people without knowledge on how food is prepared in the home. If no one was home at the selected fifth house, the next house was then selected (sixth).

### Data collection

A pretested structured questionnaire adapted from a previously published study (Gong et al. 2016) was used to collect data. Four research assistants were trained by the researchers on the contents of the questionnaire and interviewing techniques. The research assistants followed the sampling procedure by visiting every fifth house and interviewing the individual responsible for food preparation in the household (food handler). After the interview, the questionnaires were checked for completeness.

### Reliability

The study methods were applied consistently and the procedures were standardised by conducting training before the commencement of data collection. The data collection tool was pretested.

### Validity

The questionnaire was designed by adapting some existing questions from a validated and reliable questionnaire used in prior research pertaining to food safety (Gong et al. 2016).

### Data analysis

The questionnaire data were coded and entered into a Microsoft Excel spreadsheet and imported to statistical software STATA 13.1 for analysis. Descriptive statistics were performed for frequencies, mean and standard deviation. The Pearson chi-square was used to test for the relationship between categorical variables. A *p*-value of less than 0.05 was considered significant.

### Ethical considerations

All participants gave their informed consent before participating in the study. The study was conducted per the Declaration of Helsinki, and the protocol was approved by Sefako Makgatho University Research Ethics Committee (SMUREC/H/263/2016: IR).

## Results

The majority (40%; *n* = 140) of the participants were between the age range of 55 and 91 years. About 75% (*n* = 274) of the households had at least two to four adults living in the home, with 70% (*n* = 196) of households having one adult employed. The majority (66%; *n* = 227) of the participants had secondary school education (Table 1).

**TABLE 1:** Participants’ characteristics.

Characteristics	*n*	%
Male	96	28
Female	243	72
20–34 years	90	26
35–44 years	70	20
45–54 years	51	14
55–91 years	140	40
One adult	53	15
Two to four adults	274	75
Five to eight adults	38	10
One adult employed	196	70
Two to three adults employed	80	29
Four to five adults employed	3	1
No primary education	2	1
Primary education	59	17
Secondary education	227	66
Tertiary education	56	16

### Self-reported handwashing practices

As reported in Table 2, the majority (81%; *n* = 294) of the participants had best practice of washing hands before handling food and 24% (*n* = 85) had poor practices of not washing their hands after touching their face, hair, nose or mouth while handling food.

**TABLE 2:** Self-reported handwashing practices.

Practices	Response									
	Never		Rarely		Sometimes		Often		Always	
	*n*	%	*n*	%	*n*	%	*n*	%	*n*	%
Wash hands before handling food	0	0	4	1	41	11	26	7	294	81
Wash hands after handling food	8	2	7	2	43	11	28	8	281	77
Wash hands after using the toilet	2	1	0	0	8	2	22	6	331	91
Wash hands after touching face, hair, nose or mouth while handling food	35	10	50	14	109	30	29	8	142	38
Wash your hands using soap	10	3	15	4	80	22	28	8	226	63
Wash your hands using warm water	99	29	55	16	81	24	11	3	97	28

### Food preparation surfaces and utensils

The findings in Table 3 show that 38% (*n* = 139) of the participants have poor practice of using the same preparation surfaces or utensils for meat and vegetables without washing the utensils or surfaces in between. Sixty-nine percent of participants (*n* = 254) had the best practice of washing the cooking surfaces or utensils with clean warm soapy water after handling raw meat or chicken or fish (Table 3).

**TABLE 3:** Food preparation surfaces and utensils.

Variables	Response									
	Never		Rarely		Sometimes		Often		Always	
	*n*	%	*n*	%	*n*	%	*n*	%	*n*	%
Use wooden cutting board when cutting meat, chicken and/or fish	78	22	13	4	60	17	23	6	184	51
Wash preparation surfaces and utensils with clean warm soapy water after handling raw meat, chicken and/or fish	23	6	6	2	69	19	15	4	254	69
Use the same preparation surfaces and utensils used to cut meat for vegetables without washing with warm soapy water in between	14	4	9	3	75	20	10	3	129	35

### Thawing of meat, chicken and fish

Table 4 shows that 34% (*n* = 122) of the participants do not leave frozen meat, chicken or fish to thaw at room temperature or in the sun; 50% (*n* = 184) do not use microwave to thaw frozen meat, chicken and fish before cooking. Fifty-two percent (*n* = 191) never used the best practice of thawing meat, chicken or fish in cold tap running water. One hundred and sixty-four participants (45%) did not leave frozen meat, chicken or fish to thaw in a bowl with cold standing water without changing it in 30-min intervals.

**TABLE 4:** Thawing of meat, chicken and fish.

Variables	Response									
	Never		Rarely		Sometimes		Often		Always	
	*n*	%	*n*	%	*n*	%	*n*	%	*n*	%
When you want to cook frozen meat, chicken and/or fish, do you leave it to thaw at room temperature or in the sun?	122	34	29	8	83	23	31	8	96	27
When you want to cook frozen meat, chicken and/or fish, do you use microwave to thaw it?	184	50	21	6	103	28	21	6	35	10
When you want to cook frozen meat, chicken and/or fish, do you leave it to thaw in cold tap running water?	173	47	18	5	102	28	26	7	46	13
When you want to cook frozen meat, chicken and/or fish, do you leave it to thaw in a bowl with cold standing water without changing it in 30-mins intervals?	164	45	12	3	108	30	28	8	53	14

### Relationship between gender, age, education level and food safety hygiene practices

Table 5, Table 6 and Table 7 present the findings of the Pearson chi-square test on the relationship between gender, age and education level with the food handlers’ food safety hygiene practices. Table 5 indicates a statistically significant relationship (*p* < 0.05) between males and females, with females more likely to wash hands before handling food.

**TABLE 5:** Relationship between gender and food safety hygiene practices.

Hygiene practices	Gender				*p*
	Male		Female		
	*n*	%	*n*	%	
**Washing hands before handling food**					
Never	0	0	0	0	0.01*
Rarely	0	0	4	2	-
Sometimes	9	10	27	11	-
Often	13	14	11	4	-
Always	72	76	201	83	-
**Washing of hands after touching hair, face, nose or mouth while handling food**					
Never	15	16	20	8	0.06
Rarely	5	5	31	13	-
Sometimes	35	37	72	30	-
Often	8	8	21	9	-
Always	33	34	97	40	-
**Washing of preparation surfaces and utensils with clean soapy water after handling raw meat, chicken and/or fish**					
Never	6	6	17	7	0.20
Rarely	3	3	3	1	-
Sometimes	25	26	42	17	-
Often	2	2	12	5	-
Always	60	63	169	70	-
**Use same preparation surfaces/utensils used to cut meat for vegetables without washing in between**					
Never	29	30	105	43	0.07
Rarely	1	1	8	3	-
Sometimes	26	27	43	18	-
Often	2	2	8	3	-
Always	38	40	79	33	-

* , *p*-value < 0.05 is statistically significant

**TABLE 6:** Relationship between age and food safety hygiene practices.

Hygiene practices	Responses by the age of participants								*p*
	20–34 years		35–44 years		45–54 years		55–91 years		
	*n*	%	*n*	%	*n*	%	*n*	%	
**Washing hands before handling food**									
Never	0	0	0	0	0	0	0	0	0.00*
Rarely	0	0	0	0	0	0	4	3	
Sometimes	15	17	8	11	11	23	6	4	
Often	10	11	6	9	4	8	6	4	
Always	65	72	56	80	34	69	124	89	
**Washing of hands after touching hair, face, nose or mouth while handling food**									
Never	13	14	7	10	10	20	3	2	0.00*
Rarely	15	17	11	16	5	10	17	12	
Sometimes	30	33	17	24	11	22	45	33	
Often	8	9	6	9	9	17	5	4	
Always	24	27	29	41	16	31	68	49	
**Washing of preparation surfaces and utensils with clean soapy water after handling raw meat, chicken and/or fish**									
Never	8	9	1	1	6	12	8	6	0.36
Rarely	2	2	1	1	0	0	3	2	
Sometimes	10	11	14	2	11	21	32	23	
Often	5	6	2	3	2	4	5	3	
Always	65	72	52	75	32	63	92	66	
**Use same preparation surfaces and and/or utensils used to cut meat for vegetables without washing in between**									
Never	33	37	28	40	21	41	57	41	0.34
Rarely	1	1	0	0	0	0	4	3	
Sometimes	17	19	14	20	12	24	30	21	
Often	6	6	0	0	0	0	4	3	
Always	33	37	28	40	18	35	45	32	

* , *p*-value < 0.05 is statistically significant.

**TABLE 7:** Relationship between education level and food safety hygiene practices.

Hygiene practices	Responses by education level								*p*
	No formal education		Primary education		Secondary education		Tertiary education		
	*n*	%	*n*	%	*n*	%	*n*	%	
**Washing hands before handling food**									
Never	0	0	0	0	0	0	0	0	0.00*
Rarely	0	0	0	0	4	2	0	0	
Sometimes	0	0	1	2	25	11	15	28	
Often	0	0	0	0	22	10	4	7	
Always	2	100	58	98	176	77	35	65	
**Washing of hands after touching hair, face, nose or mouth while handling food**									
Never	0	0	1	2	26	11	4	7	0.27
Rarely	0	0	7	12	32	14	7	13	
Sometimes	1	50	18	30	60	27	23	41	
Often	0	0	6	10	16	7	7	13	
Always	1	50	27	46	92	41	15	26	
**Washing of preparation surfaces and utensils with clean soapy water after handling raw meat, chicken and/or fish**									
Never	0	0	5	6	14	6	4	7	0.02*
Rarely	0	0	0	0	2	1	4	7	
Sometimes	0	0	13	17	36	16	10	18	
Often	1	50	21	27	10	4	2	4	
Always	1	50	39	50	165	73	36	64	
**Use same preparation surfaces and utensils used to cut meat for vegetables without washing in between**									
Never	0	0	23	39	90	40	22	39	0.00*
Rarely	1	50	1	2	5	2	0	0	
Sometimes	0	0	16	27	35	15	16	28	
Often	0	0	0	0	9	4	1	2	
Always	1	50	19	32	88	39	17	30	

* , *p*-value < 0.05 is statistically significant.

Table 6 illustrates a statistically significant relationship (*p* < 0.05) between age and the following food safety hygiene variables, namely, washing hands before handling food and washing of hands after touching hair, face, nose or mouth while handling food, whereby older food handlers had better practices as compared to other age groups.

Table 7 indicates a significant relationship (*p* < 0.05) between education level and the following food safety hygiene variables, namely, washing hands before handling food, washing of preparation surfaces and utensils with clean soapy water after handling raw meat, chicken and/or fish, and lastly using the same preparation surfaces and utensils used to cut meat for vegetables without washing in between. The more educated the food handlers, the better their practices regarding food safety hygiene.

## Discussion

The current study assessed food safety practices of households in Ga-Rankuwa, Tshwane. The majority of the participants reported always washing their hands before and after handling food. Similarly, a study by Langiano et al. (2012) reported that the majority of participants reported to always wash their hands with soap and water after handling raw meat. The current study’s findings, however, were self-reported, which means that participants could over-report or only report on desirable behaviours. Furthermore, Ruby et al. (2019) reported that people may be very knowledgeable about handwashing, but that knowledge may not be put into practice. Handling food without proper handwashing predisposes individuals to diseases which could be prevented (Centers for Disease Control and Prevention 2020). The researchers are of the view that washing of hands could have improved due to the coronavirus disease 2019 (COVID-19) pandemic where peoples’ behaviour was influenced (Mucinhato et al. 2012). Kasza et al. (2022) observed that at least once among 15 households, the food handler did not properly wash their hands during food handling even after touching raw chicken or after sneezing or coughing. In the current study, age had a positive influence (*p* < 0.05) on the washing of hands before handling food and after touching their hair, face, nose or mouth. Older food handlers were more likely to wash their hands during handling.

More than 50% of respondents in the current study reported always using a wooden cutting board for food preparation. Similarly, Mucinhato et al. (2012) reported that individuals were more likely to use wooden cutting boards, thus increasing the potential risk of cross-contamination. Moreover, Mucinhato et al. (2012) stated that the home food preparation environment can be a serious breeding ground for foodborne diseases. Studies by Kasza et al. (2022) and Møretrø et al. (2021) in six European countries reported on the microbiological analysis of household chicken samples and found that most of the samples were contaminated with *Salmonella* and *Campylobacte*r which were transmitted to the cutting board during meal preparation. Contamination with *Salmonella* can also occur after cooking through contact with surfaces and containers of previously contaminated cooking utensils from the uncooked meat (Gallo et al. 2020). In a study by Langiano et al. (2012), most participants believed it was not necessary to clean and disinfect cutting boards between preparing different foods.

According to the current study’s findings, the majority of participants (69%) always wash preparation dishes and utensils with clean, warm, soapy water before handling raw meat, chicken or fish. Thirty-nine percent of the participants reported that they never use the same preparation surfaces or utensils to cut meat and vegetables without washing with warm soapy water in between.

The transmission of foodborne diseases is aggravated by unsafe food handling practices (Dagne et al. 2019). A good level of food safety practice of mothers was associated with good levels of educational status (Dagne et al. 2019). Thus, those with higher education are able to read print material even though they may not implement the correct behaviour in food safety. Food safety training is therefore important for individuals to practise safe food handling.

The current study found a significant relationship between the education level of the food handlers and their food safety and food hygiene practices. Similarly, Ababio and Adi (2012) also reported a significant relationship between the education level of food handlers in Kumasi, Ghana, and their food hygiene practices. Therefore, education programmes targeting food handlers in Ga-Rankuwa could be effective in improving their food handling practices and subsequently reducing the risk of foodborne diseases in the home.

Many South Africans depend largely on raw and semi-processed foods and there is a high possibility of cross-contamination between these foods and the kitchen surfaces where they are processed (Mkhungo et al. 2018). Therefore, food handlers in the home have the important responsibility of preventing food contamination during the preparation and distribution of food.

Less than half (45%) of the participants in the current study reported never leaving frozen meat, chicken and fish to thaw in a bowl with cold water before cooking and more than 50% thaw at room temperature. Similarly, Langiano et al. (2012) found that it was common for participants to thaw meat and fish at room temperature. Furthermore, Leygonie, Britz and Hoffman (2012) discovered that the risk of thawing frozen chicken in a bowl of water or over the counter is that the increased water activity combined with the relatively warm temperature is favourable for pathogen growth. To avoid rapid microbial growth, meat, chicken or fish should be thawed in a refrigerator (Food Safety and Inspection Service, United States Department of Agriculture 2013). Food hygiene education, targeting households, should be prioritised as a strategy to prevent foodborne illnesses.

### Strengths and limitations

One of the strengths of the study was the use of a questionnaire which had been used in a previously published study and that was adapted for the current study. Also following the pilot study, questions were rephrased if they were ambiguous to the participants. This ensured the validity of the questionnaire. There is also a likelihood of over-, under- or misreporting of findings in self-reported studies. The study was conducted in one area in Tshwane and can be replicated in other areas. Participant responses may be skewed due to their socially desirable ability to provide preferred answers more than real experiences (Grimm 2010).

### Implication of the study

The study focused on understanding the practices of food handlers in households. Thus, providers of training can be informed through this study to enable them to put in place appropriate strategies to improve individuals’ understanding on food safety and proper food hygiene. Future research can extend this work by examining the actual behaviour of food handlers in households during food preparation.

## Conclusion

Food safety concerns include those involved in food preparation, including food handlers, as well as sanitation of the environment and the equipment used. Food safety and a person’s diet have an impact on their health and nutritional intake. However, the challenge is that food preparers in households are not formally trained in food safety. What people do in households is what they understand to be best practice learned from other family members.
